# Community-Based Malaria Testing Reduces Polypharmacy in a Population-Based Survey of Febrile Illness in Western Kenya

**DOI:** 10.3389/ijph.2022.1604826

**Published:** 2022-08-25

**Authors:** Jeremiah Laktabai, Alyssa C. Platt, Elizabeth Turner, Indrani Saran, Joseph Kipkoech, Diana Menya, Wendy Prudhomme O’Meara

**Affiliations:** ^1^ School of Medicine, Moi University, Eldoret, Kenya; ^2^ Academic Model Providing Access to Healthcare (AMPATH), Eldoret, Kenya; ^3^ Duke Global Health Institute, Duke University, Durham, NC, United States; ^4^ Department of Biostatistics and Bioinformatics, School of Medicine, Duke University, Durham, NC, United States; ^5^ School of Social Work, Boston College, Chestnut Hill, MA, United States; ^6^ Moi University School of Public Health, Eldoret, Kenya

**Keywords:** antibiotics, malaria, antimalarials, fever, polypharmacy, malaria testing

## Abstract

**Objective:** The objective was to describe the relationship between the location of care, the malaria test result, and the type of medicine consumed for the fever, and to determine whether community-based access to malaria testing reduced polypharmacy**.**

**Methods:** This is a secondary analysis of a cluster-randomized trial of an intervention designed to increase diagnostic testing and targeting of Artemesinin Combined Therapies (ACTs). Data collected at baseline, 12, and 18 months were analyzed to determine the impact of diagnostic testing on drug consumption patterns among febrile individuals.

**Results:** Of the 5,756 participants analyzed, 60.1% were female, 42% were aged 5–17 years, and 58.1% sought care for fever in a retail outlet. Consumption of both ACT and antibiotics was 22.1% (*n* = 443/2008) at baseline. At endline, dual consumption had declined to 16.6%. There was reduced antibiotic consumption among those testing positive for malaria (39.5%–26.5%) and those testing negative (63.4%–55.1%), accompanied by a substantial decline in ACT use among malaria-negative participants.

**Conclusion:** Diagnostic testing for malaria reduces dual consumption of ACTs and antibiotics, especially among those testing outside the formal healthcare sector.

## Introduction

Rational use of medicines requires that “patients receive medications appropriate to their clinical needs, in doses that meet their own individual requirements, for an adequate period of time, and at the lowest cost to them and their community” [1]. Nevertheless, in many low and middle income countries, it is estimated that more than 50% of all medicines are inappropriately prescribed [2]. Irrational use of medication includes polypharmacy which is the prescription of more medicines than is clinically indicated, inappropriate self-medication, and use of antibiotics for non-bacterial infections [3, 4].

In settings where access to laboratory investigations for fever is limited, empiric prescription of antibiotics and antimalarials is common due to the inability to discriminate the etiologies of fever. This can lead to unnecessary prescriptions and the potential consequences include drug pressure risking resistance, adverse drug events, and a higher cost of care [5, 6].

Improved access to diagnostic testing for malaria can potentially reduce unnecessary antimalarial overuse [7] as well as minimize co-prescription of antibiotics and antimalarials for confirmed malaria cases. Conversely, it can lead to a paradoxical increase in the use of antibiotics for non-malarial fevers [8, 9].

Many fevers are treated in the informal sector without access to skilled health workers [10–12]. Self-medication is also common [13–15], and these lend themselves to inappropriate uses of medications. However, empiric treatment and polypharmacy are also common in the formal health sector, driven in part by the lack of diagnostics and by the health workers’ beliefs (or uncertainty) about the local etiology of fever [16].

As part of a larger study involving access to malaria rapid diagnostic testing at the community level, we examined the impact of malaria diagnostic testing on drug consumption patterns. The objective of diagnostic testing is to increase certainty about the cause of illness and treat the underlying cause of fever more accurately. We hypothesized that increased access to diagnostic testing should reduce uncertainty and thus reduce overtreatment with more than one antimicrobial agent.

## Methods

### Study Design

A cluster-randomized controlled trial (cRCT) was conducted in 32 community units (CUs, i.e., clusters) in western Kenya (population approximately 160,000). Community units were randomly assigned to the intervention or comparison (usual care) arm. Community health workers (CHWs) offered on-demand free rapid diagnostic tests (RDTs) to febrile individuals in the intervention arm. A voucher to purchase a WHO-qualified artemisinin combination therapy (ACT) was then issued to the febrile individual contingent on a positive test result. The voucher provided ACT at a reduced fixed price in nearby retail outlets. Between July 2015 and May 2017, 32,404 participants were tested for malaria, and 10,870 vouchers were issued. The goal was to draw individuals who would typically seek care in the retail sector to have a diagnostic test performed by a community health worker (CHW) before purchasing drugs. Outcomes were measured using three community-based surveys of a random sample of people with a recent fever at 6-, 12- and 18-month follow-up after the initial baseline community-based survey, with the guardian responding on behalf of a child under the age of 18. We found that at the final follow-up time point of 18-months, the intervention substantially increased testing, with 55.0% of febrile individuals in the intervention area taking a malaria test versus 44.7% in the comparison am (risk ratio [RR] = 1.25, 95% CI: 1.09–1.44). Additional primary and secondary results of the cRCT can be found elsewhere [17].

### Study Population and Randomization

The cRCT was conducted in 3 subcounties in Western Kenya (Webuye West, Webuye East, and Kiminini). Each subcounty is divided into community units (CUs). A CU is a geographically defined unit including approximately 1,000 households served by 10–22 CHWs. All CUs with active, pre-existing CHWs in each subcounty were eligible to participate in the study (10 Webuye East, 8 Webuye West, and 14 Kiminini). Randomization occurred at the CU level, stratified by sub-county and the CU-level presence or absence of a public health facility offering malaria diagnostic testing. Each stratum contained an even number of CUs that were assigned equally to the comparison or intervention arm. Additional details about the study population, including patient flow, can be found elsewhere [17, 18].

### Intervention

In the intervention CUs, CHWs were trained to perform malaria rapid diagnostic tests (RDTs) and to offer free testing to individuals in their CUs. Any individual older than one year and reporting a fever or malaria-like illness in the previous 48 h could present to the CHW closest to them for a free RDT. If the test was positive, the CHW would provide a voucher. Local medicine retailers serving residents of the intervention clusters were enrolled in the study. Study participants could redeem their vouchers for a discounted quality-assured artemether-lumefantrine (AL; “conditional subsidy”). Quality-assured AL is the first-line ACT recommended for uncomplicated malaria by the Government of Kenya. All participants received a referral note with the test results, whether positive or negative and could present it to a facility of choice for further evaluation. The facilities within the study catchment were aware of the intervention and agreed to honor the test results from the community. The CHWs did not dispense any drugs; their role was limited to testing and referral. Comparison CUs continued to have access to standard healthcare, including government health facilities, private health facilities, and pharmacies or retail medicine outlets. Additional information about the intervention design can be found elsewhere [18].

### Outcome Measures for the Current Secondary Data Analysis

This is a secondary analysis of data from the above study. Given that the original intervention succeeded in increasing malaria testing and targeting of ACTs, this secondary analysis seeks to determine whether the intervention substantially changed the overall patterns of drug consumption and polypharmacy in febrile individuals. For the current study, data collected at baseline and after 12 and 18 months of follow-up were analyzed. Data from the 6-month follow-up wave were excluded because the intervention was expected to reach full effect after a year of implementation.

The two primary outcomes for the current study were the types of drugs taken and the combination of drugs taken. Data were obtained from a pre-populated list of key drugs as well as free text options. “Types of drugs taken” were classified according to 12 categories (AL; Other ACT; Non- ACT antimalarial (Quinine, SP, other antimalarial); Antibiotic (Amoxyl/Seprin/Cipro/Norfo); Other Antibiotic; Allergy/cough/asthma; Deworm; Painkiller; Stomach/GI; Traditional/herbal; Vitamin; Other). “Combination of drugs taken” was based on the four possible combinations of use versus non-use of two drug classes, namely ACTs and antibiotics, where the combined use of ACTs (AL and Oother ACT) and antibiotics is referred to as polypharmacy. The secondary outcomes were consumption of an ACT and the place of testing for those who had a malaria test.

Details on the derivation of the primary and secondary outcomes are in Supplementary S1.

### Statistical Analysis

To assess general trends and explore factors that were associated with different treatment decisions, we conducted a descriptive analysis for outcomes of interest by stratifying on key covariates: treatment arm (comparison and intervention), wave (baseline and follow-up, with follow-up defined as 12- and 18-month waves combined), test behavior (took a malaria test versus not), and test result (positive, negative). Regression analysis was used to assess the impact of the intervention on the polypharmacy outcome. While those regression analyses provided overall inference on the difference between arms in drug consumption patterns, we were also interested in the potential roles of location of testing and test result on drug consumption behavior. Therefore, we did further descriptive analysis, stratifying means and frequencies by location of testing. Descriptive analysis was performed using R version 3.4.1 software, while regression analysis was completed in SAS 9.4 using PROC GLIMMIX. All the drugs an individual took for their illness were aggregated across all the treatment seeking steps.

The regression analyses used to assess the overall impact of the intervention on polypharmacy at the pooled 12- and 18-month follow-up period were planned within the generalized linear mixed-effects modeling framework, using a random intercept for CU. However, in practice, convergence issue precluded the inclusion of random intercepts due to low variability in the outcome between CUs. A logit-link and multinomial distribution were used for the four-level drug combination outcome, for which parameters were exponentiated to provide odds ratios for each drug combination compared to the reference level of “neither antibiotics nor ACT.” Each model included fixed effects for strata (interaction between sub-county and health-facility) in order to account for the stratified, cluster-randomized design. Each model was also adjusted for participant age and gender, respondent education and socio-economic status (wealth percentile based on a household asset index following standard methods), and whether the participant indicated they still felt sick in order to estimate adjusted odds ratios (AOR) Though the multinomial outcome involved three tests for the intervention effect, the main outcome of interest was polypharmacy (combined use of ACT and antibiotics), thus no corrections for multiple comparisons were performed. To supplement our hypothesis testing on the drug combination outcome, we also performed a joint test on a null hypothesis that there was no difference between intervention and comprison arms in the odds of taking any of the ACT and antibiotic drug combinations versus the reference level of no ACT and no antibiotics.

## Results

A total of 5,756 individuals with fever in the last one month participated in the surveys included in this analysis: 2,017 at baseline, 1,812 at 12 months, and 1,927 at 18 months.

For the 2,017 individuals who participated in the pre-intervention baseline survey (977 in the comparison and 1040 in the intervention arms, respectively), demographic characteristics in the comparison and intervention arms were similar and can be found in O’Meara et al. [15]**.** In brief, the majority of respondents were female (60.1%), most were under the age of 18 (63.9%), and 42% had not completed primary school. Care seeking and malaria testing at baseline are described in Table 1. Half sought care at a health facility (52.7%), however, most did not have a test for malaria (57.4%) and, overall, among those that tested, malaria positivity was reported in a large majority (84.6% and 79.3% in the comparison and intervention arms, respectively).

**TABLE 1:** Care seeking characteristics of the study participants by study arm at baseline, Western Kenya, 2015–2017.

	Comparison	Intervention	Total
n	977	1,040	2,017
Treatment Seeking, n(%)			
No treatment	3 (0.3)	10 (1.0)	13 (0.6)
Treatment only at home	59 (6.0)	75 (7.2)	134 (6.6)
Treatment at any health facility	523 (54.6)	517 (50.8)	1040 (52.7)
Visited a private health facility	156 (16.3)	157 (15.4)	313 (15.8)
Visited a public health facility	384 (40.1)	374 (36.8)	758 (38.4)
Visited a shop/pharmacy	538 (56.2)	610 (60.0)	1148 (58.1)
Sought multiple types of treatment^a^	408 (41.8)	434 (41.7)	842 (41.8)
Took a malaria test, n (%)	436 (44.8)	421 (40.6)	857 (42.6)
Malaria Test Result, n (%)			
Positive	369 (84.6)	334 (79.3)	703 (82.0)
Negative	67 (15.4)	83 (19.7)	150 (17.5)
Don’t remember/Don’t know	0 (0.0)	4 (1.0)	4 (0.5)

a Individual reported treatment seeking as more than one of the following options: treatment at home, treatment by CHW, treatment at private health facility, treatment at a public health facility, visit to shop/pharmacy, visited traditional healer, visited religious/cultural healers, other.

### Drug Consumption by Malaria Testing Status

Almost all ACT consumed was AL (97.6%, 4021/4119). At baseline, 44.8% and 52.4% of those reporting a negative malaria test result took AL, the first-line therapy for malaria, in the comparison and intervention arm, respectively (Table 2). A much higher proportion of those testing positive took AL (86.4% comparison arm v. 86.1% in the intervention arm). By follow-up, AL consumption among those testing negative in the intervention arm was nearly 20-percentage points lower than in the comparison arm (36.1% vs. 53.6%). AL consumption among positives was slightly higher in the intervention arm compared to the comparison arm (90.4% vs. 86.4%). Overall, the comparison arm saw little change in AL consumption across testing categories by the follow-up period. Among those without a test, AL consumption remained high and stable across time and groups; between 64.4% and 74.4% of untested respondents took AL.

**TABLE 2:** Number and types of drugs categories taken for a febrile episode by treatment arm and by malaria test result stratified by baseline and pooled 12- and 18-month follow-up waves, Western Kenya, 2015–2017.

	Comparison						Intervention					
	Baseline			Follow-up			Baseline			Follow-up		
	Negative	No test	Positive	Negative	No test	Positive	Negative	No test	Positive	Negative	No test	Positive
n	67	538	369	84	1022	679	83	617	334	152	887	779
Total Drugs Types Taken^a^ mean (SD)	2.16 (0.81)	1.74 (0.79)	2.28 (0.86)	1.92 (0.84)	1.76 (0.71)	2.16 (0.72)	2.13 (1.00)	1.68 (0.78)	2.24 (0.86)	1.54 (0.78)	1.62 (0.75)	2.12 (0.65)
Type of Drug Taken												
Antibiotics n (%)	45 (67.2)	134 (26.4)	148 (40.3)	59 (70.2)	212 (22.0)	225 (33.2)	52 (63.4)	141 (24.5)	131 (39.5)	81 (55.1)	183 (22.3)	206 (26.5)
ACT n (%)	30 (44.8)	343 (67.5)	331 (90.2)	45 (53.6)	727 (75.4)	609 (90.0)	45 (54.9)	384 (66.7)	293 (88.3)	54 (36.7)	540 (65.8)	718 (92.3)
AL n (%)	30 (44.8)	335 (65.9)	317 (86.4)	45 (53.6)	717 (74.4)	585 (86.4)	43 (52.4)	378 (65.6)	286 (86.1)	53 (36.1)	529 (64.4)	703 (90.4)
Non-ACT antimalarial n (%)	7 (10.4)	35 (6.9)	78 (21.3)	3 (3.6)	54 (5.6)	122 (18.0)	8 (9.8)	57 (9.9)	81 (24.4)	4 (2.7)	64 (7.8)	104 (13.4)
Analgesics n (%)	63 (94.0)	452 (89.0)	325 (88.6)	76 (90.5)	882 (91.5)	610 (90.1)	72 (87.8)	487 (84.5)	284 (85.5)	122 (83.0)	734 (89.4)	717 (92.2)
Other n (%)	1 (1.5)	0 (0.0)	6 (1.6)	3 (3.6)	0 (0.0)	1 (0.1)	0 (0.0)	0 (0.0)	2 (0.6)	1 (0.7)	0 (0.0)	1 (0.1)
Drug Combinations												
ACTs with Antibiotics n (%)	17 (25.4)	81 (15.9)	134 (36.5)	27 (32.1)	142 (14.7)	198 (29.2)	27 (32.9)	72 (12.5)	112 (33.7)	21 (14.3)	104 (12.7)	176 (22.6)
ACTs, No Antibiotics n (%)	13 (19.4)	262 (51.6)	197 (53.7)	18 (21.4)	585 (60.7)	411 (60.7)	18 (22.0)	312 (54.2)	181 (54.5)	33 (22.4)	436 (53.1)	542 (69.7)
Antibiotics, No ACTs n (%)	28 (41.8)	53 (10.4)	14 (3.8)	32 (38.1)	70 (7.3)	27 (4.0)	25 (30.5)	69 (12.0)	19 (5.7)	60 (40.8)	79 (9.6)	30 (3.9)
Neither ACT nor Antibiotics n (%)	9 (13.4)	112 (22.0)	22 (6.0)	7 (8.3)	167 (17.3)	41 (6.1)	12 (14.6)	123 (21.4)	20 (6.0)	33 (22.4)	202 (24.6)	30 (3.9)

a Number of types of drugs taken for each participant is defined as the sum of binary indicators for each drug. With the possible range being 0–12 and the categories being the following: AL; ACT (AL or other ACT); Non- ACT antimalarial (Quinine, SP, other antimalarial); Antibiotic (Amoxyl/Seprin/Cipro/Norfo); Other Antibiotic; Allergy/cough/asthma; Deworm; Painkiller; Stomach/GI; Traditional/herbal; Vitamin; Other.

Declining ACT consumption among malaria-test negative respondents was not accompanied by an increase in antibiotic consumption. On the contrary, the intervention arm saw a slight decrease in antibiotic use across all three categories (untested, positive, negative), with the largest decline seen in those testing positive (39.5% vs. 26.5%). Slightly more than half of those with a negative test reported taking an antibiotic in the intervention arm at follow-up (55.1%) compared to 70.2% in the comparison arm.

Use of non-ACT antimalarials was not common. At baseline, about 20%–24% of those with a positive test reported taking a non-ACT antimalarial. This saw a relative decline of nearly 50% among respondents in the intervention arm at follow-up (13.4%). Most non-ACT antimalarials were artemisinin injections, followed by quinine tablets.

### Drug Combinations

The use of antibiotics in combination with antimalarials according to test results at baseline and follow-up in both arms is summarized in Table 2. At baseline, of those testing negative for malaria, 25.4% in the comparison and 32.9% in the intervention arm took both an ACT and an antibiotic. Between 33.7% and 36.5% of those with a positive test also used both antibiotics and ACT, but among untested respondents, the percentage was lower (12.5%–15.9%). By the follow-up time point, the proportion of test-negative clients taking both an ACT and an antibiotic in the intervention arm saw a relative decline of 57%, from 32.9% down to 14.3%. In contrast, little change was observed in combination treatment for those with a positive test and those without a test.

We compared the use of ACT with antibiotics, ACT alone, antibiotics alone, and no antimicrobials between the intervention and the comparison arm at follow-up (pooled 12 and 18 month surveys, Table 3). There is evidence for reduced polypharmacy (i.e., reduced consumption of ACT with antibiotics) in the intervention arm compared to the control arm with proportions of 18.9% vs. 22.5%, respectively (AOR = 0.694, 95%CI: 0.547, 0.880, *p* = 0.002). There was little evidence of differences in use of antibiotics alone or ACT alone between arms, but these comparisons may have been limited by small cell size, particularly for the category of antibiotics alone.

**TABLE 3:** Adjusted model estimated between-arm differences comparing intervention versus comparison arm in number of types of drugs^a^ and drug combinations^b^ consumed at pooled 12- and 18-months follow-up waves in Western Kenya 2015–2017.

Drug combinations (N = 3,540)	Sample proportions (%)		Odds ratio (95% CI)	*p*-value
	Comparison	Intervention		
ACTs with Antibiotics	608 (22.5)	526 (18.9)	0.69 (0.55, 0.88)	0.003
Antibiotics, No ACT	234 (8.6)	290 (10.4)	1.02 (0.77, 1.35)	0.904
ACT, No Antibiotics	1500 (55.4)	1541 (55.4)	0.84 (0.68, 1.03)	0.087
Neither ACT nor Antibiotics	366 (13.5)	427 (15.3)	Reference	—
Type III joint test in intervention parameter^c^				0.004

a Number of types of drugs taken for each participant is defined as the sum of binary indicators for each drug, possible categories are: AL; Other ACT; Non- ACT antimalarial (Quinine, SP, other antimalarial); Antibiotic (Amoxyl/Seprin/Cipro/Norfo); Other Antibiotic; Allergy/cough/asthma; Deworm; Painkiller; Stomach/GI; Traditional/herbal; Vitamin; Other; Analysis by linear regression with random intercepts for community unit (CU).

b Drug combinations regression estimated with multinomial logistic regression, models exclude adjustment for clustering of outcomes by CU, due to convergence issues with random intercept models.

c Joint test that intervention effect = 0 for all outcomes.

### Drug Consumption Behavior by Location of Malaria Testing

It is instructive to look at the drug consumption patterns by testing location to understand how the health-seeking context influences treatment decisions. For this analysis, we pooled time periods and arms to avoid very small cell sizes. Nearly all participants (*n* = 294, 98.0%) testing positive at the CHW consumed AL (Table 4), with the second highest AL consumption rate occurring amongst participants testing positive at a public health facility (N = 1135, 90.7%). Fewer participants consumed AL after a positive test at a private facility (N = 457, 76.9%) and many of those took non-ACT antimalarials. Most malaria positives tested at the CHW and consuming AL reported obtaining their AL at a retail shop with their voucher (271, 93.4%).

**TABLE 4:** Location of malaria testing and drug consumption, pooled over study arm and the baseline, 12-month and 18-month follow-up waves in Western Kenya 2015–2017.

	No test	Tested at CHW		Tested at private		Tested at public	
		Negative	Positive	Negative	Positive	Negative	Positive
Observations	3064	85	301	40	597	259	1255
Take AL?	1959 (68.3)	35 (43.8)	294 (98.0)	16 (40.0)	457 (76.9)	118 (45.7)	1135 (90.7)
Drug Combinations							
ACTs with Antibiotics n (%)	399 (13.9)	8 (10.0)	16 (5.3)	12 (30.0)	186 (31.3)	71 (27.5)	418 (33.4)
ACTs, No Antibiotics n (%)	1595 (55.6)	27 (33.8)	278 (92.7)	4 (10.0)	310 (52.2)	50 (19.4)	737 (58.9)
Antibiotics, No ACTs n (%)	271 (9.4)	26 (32.5)	0 (0.0)	12 (30.0)	39 (6.6)	107 (41.5)	50 (4.0)
Neither ACT nor Antibiotics n (%)	604 (21.1)	19 (23.8)	6 (2.0)	12 (30.0)	59 (9.9)	30 (11.6)	47 (3.8)
Total Drug Types Taken (SD)	1.70 (0.75)	1.51 (0.78)	1.95 (0.37)	1.75 (1.10)	2.11 (0.79)	1.99 (0.86)ET	2.27 (0.78)
Antibiotics (n, %)	670 (23.4)	34 (42.5)	16 (5.3)	24 (60.0)	225 (37.9)	178 (69.0)	468 (37.4)
Analgesics (n, %)	2555 (89.1)	67 (83.8)	278 (92.7)	33 (82.5)	512 (86.2)	232 (89.9)	1140 (91.1)
ACT (n, %)	1994 (69.5)	35 (43.8)	294 (98.0)	16 (40.0)	496 (83.5)	121 (46.9)	1155 (92.3)
Non-ACT antimalarial	210 (7.3)	1 (1.2)	7 (2.3)	1 (2.5)	110 (18.5)	20 (7.8)	267 (21.3)

Among respondents who tested negative at a public health facility, 45.7% took AL, and most got their AL at the facility or with a prescription from the facility. Similar outcomes are observed in private facilities. Of those who tested negative from the CHW, 43.8% took AL, and more than half obtained AL over-the-counter. Almost eighty percent of untested respondents who took AL purchased their AL over-the-counter.

Antibiotic consumption was relatively rare in malaria positives testing at the CHW (N = 16, 5.3%) versus positives who tested at private (N = 225, 37.9%) and public facilities (N = 468, 37.4%). Similarly, 42.5% (N = 34) of respondents with a negative test from the CHW consumed antibiotics versus 60.0% (N = 24) and 69.0% (N = 178) at private and public health facilities, respectively. Very few malaria-positive respondents who tested at the CHW took ACT with antibiotics (5.3%), again compared to very high combination treatment in the public and private facilities (33.4% and 31.3%, respectively).

Overall, the average number of drugs dispensed to a patient was highest in the public sector, followed by the private formal sector. Respondents with a negative test or no test consistently took fewer types of drugs than those with a positive test (Table 2).

## Discussion

The choice of the place of seeking care for a fever episode is a balance of multiple factors such as the cost of care, distance, previous experiences and expectations of the patients and the caregivers. The study confirms that retail medicine shops are the preferred first choice for care for fever, as has been demonstrated in numerous previous studies [13, 19, 20].

The cost of first-line ACTs and the falling prevalence of malaria necessitates diagnostic testing to confirm the presence of parasites, and targeted treatment based on test results can reduce unnecessary consumption of antimalarials [7]. Less than half of respondents had a malaria test for their fever episode at baseline. This is expected given that the retail shops, where care is most sought, do not routinely perform malaria testing. Even in health facilities where testing is routine, stockouts of testing kits, and poor uptake of testing due to human resource challenges limit access to malaria testing [21–24]. In the intervention arm, where CHWs were empowered to perform free malaria testing in the community, the testing uptake for those with a fever episode increased, confirming that availability and affordability of testing are important contributors to testing uptake [25, 26].

In this study, we saw substantial shifts in the use of antimalarials and antibiotics between the intervention and comparison groups. These changes were most noticeable in the decrease in test-positive malaria cases that consumed antibiotics and the reduction in consumption of AL by test-negative fever cases. As a result of these changes, polypharmacy, or the use of the combination of ACT plus antibiotics for a fever, saw a relative decline in the intervention arm of 56.5% among test negative cases and 32.9% in test-positive fevers between baseline and followup. Comparing across arms at follow-up, the odds of polypharmacy was approximately 30% lower in the intervention arm (*p* = 0.003). We also note that the proportion of test-negative fevers consuming antibiotics also declined in the intervention arm at 18 months. However, this result cannot fully allay fears about possible increased antibiotic consumption as the test-positivity rate declines, and more test-negative fevers are seen. Overall, these improvements highlight an important role for increased testing to shift drug consumption patterns. However, they also indicate that changes in adherence to the test are required in order to reduce overconsumption of antimicrobials, and that response to a test may be influenced by the context of testing, including who is interpreting and acting on the test.

The high rates of prescription of ACTs to patients with a negative malaria test result is surprising given the emphasis on Test before Treat policy for malaria case management by the Kenya Ministry of Health and stands in contrast with other studies that show very high adherence to negative test results in country-wide surveys of Kenyan health facilities [27]. This may reflect changes in health worker confidence in malaria diagnostic testing over time or a possible Hawthorne effect introduced by data collectors’ presence during health facility surveys.

A decision on the number and type of medication taken for fever is multifactorial, including a formal prescription from a health care worker, a patient’s request, and a recommendation by a retail outlet dispenser. Retail shops clinically diagnose based on reported symptoms and recommend medications [28], based on factors such as their level of knowledge and skills, expectations by the clients, profit considerations and promotions by the pharmaceutical industry [29, 30]. Additionally, adherence to a malaria test result is influenced by many factors, including belief in performance of the test kits, existing clinical practices, patient expectations and the availability of alternative diagnosis for a non-malarial fever [6, 19, 31–34]. In our study, we demonstrate marked differences in consumption of antimicrobials depending on the location of testing. Prescription or dispensing of ACT with an antibiotic was very high in the formal health sector, but nearly absent among those tested by the CHW. Fevers who were tested by a CHW adhered narrowly to the result and antibiotic consumption among those with a negative test was low. This indicates very different drivers for drug prescription by testing location, including possibly lower confidence in testing among health care providers or more severe illness seen at the facility. Access to malaria testing and case management should ideally be part of comprehensive fever management strategy that considers more than just whether to use ACT treatment or not [25, 35, 36].

Some limitations to this analysis must be noted. First, we were unable to control for within-cluster correlation in our analysis of medication combinations, due to convergence issues caused by low variability in the outcome between Cus. However, because such correlation was low, we do not expect for this to have a large impact on standard errors. Second, inferential analysis was done broadly between arms, pooling the 12- and 18-month time points. Some of the differences between testing locations may have been a result of self-selection of clients into different groups, for instance, more severe illness attending a health facility, whereas fevers considered less severe were tested by the CHW or treated over-the-counter. Finally, though we self-reported malaria test results, we cannot know the true etiology of fever for most of the sample participants, and therefore, we cannot ultimately identify the appropriateness of the observed drug consumption patterns.

### Conclusion

We found that dual prescription of ACT with antibiotics declined following implementation of a community-based malaria diagnostic testing intervention. This decline was most prominent among those who received positive malaria test results from a CHW. Access to malaria testing and case management should ideally be part of comprehensive fever management strategy, inclusive of the retail sector.
