# Supplementary material for: Community-Based Malaria Testing Reduces Polypharmacy in a Population-Based Survey of Febrile Illness in Western Kenya
Source: Int J Public Health. 2022 Aug 25;67:1604826. doi: 10.3389/ijph.2022.1604826 (PMC9453644; doi:10.3389/ijph.2022.1604826)
Supplement: Supplementary file 1 [file DataSheet1.pdf]

International Journal of Public Health

Title: Community-based malaria testing reduces polypharmacy in a population-based survey of febrile illness in Western Kenya

S1 Text: Derivation of outcomes

The primary outcomes were the number of types of drugs consumed and the factorial combination of use versus non-use of ACTs and antibiotics. The secondary outcomes were consumption of an ACT, the source of the quality approved ACT and the place of testing for those who had a malaria test.

Individuals surveyed were asked: "Did you take any drugs for your illness?", followed by "Which medicines did you take?" Individuals were given a list of drug categories from which they could select multiple different types of medicines including: Coartem/Artefan/other AL, Quinine; SP/Fansidar/Metakelfin/Moladar; Other ACTs (e.g Duocotexin); Amoxyl; Septrin; Ciprofloxacin/Norfloxaccin; Panadol/Brufen/Hedex/Painkiller/Action/Maramoja, Don't know/Don't Remember; and Other (**S1 Table, Column 1**). Those indicating that they had taken "Other" medicines were asked specifically which medicines they took – which was subsequently entered as free text response. Common free text responses with medicines not included in the predefined list were added as new categories for the purposes of enhancing the accuracy of main outcome variables. Common free text drug types that were not included in the prespecified list included: Allergy/Cough/Asthma, Antifungal, Deworming, Other Antibiotics, SP, Stomach/GI, Traditional/Herbal, Vitamin.

Where individuals answered in free text response that they had taken a drug that was among the existing categories, their response was re-categorized as such.

Number of types of drugs

For the purposes of ascertaining the number of types of drugs consumed, answer choices and additional free text categories were collapsed to reduce redundancy of categories (Table S2, Column 2). Specifically, antimalarials were collapsed into two categories (ACT antimalarial and non-ACT antimalarial) and antibiotics were collapsed into two categories (Amoxyl/Septrin/Ciprofloxacin/Norfloxaccin and Other antibiotics). Each of the collapsed categories was represented by a binary variable set to one when an individual listed that drug category in their list of drugs taken. Categories were summed to create an overall measure that could take a value from 0 to 12.

Drug combinations

We created a second outcome measure to summarize specific drug combinations of interest, namely, the factorial combination of ACTs and antibiotics. Each response was categorized by the presence or absence of each of the two drug classes yielding four categories: No ACT or

Antibiotic, ACTs with no antibiotics, antibiotics with no ACTs, and ACTs with antibiotics (**S1 Table**, Column 3). This outcome is treated as a nominal, multinomial outcome.

S1 Table. Drug categories from survey with collapsed categories

Which medicines did you take for your current illness?		
Original Category	Collapsed (Number of Drugs)	Collapsed (Drug Combinations)
Coartem/Artefan/other ACT	ACT	ACT
Quinine	Non-ACT Antimalarial	
SP/Fansidar/Metakelfin/Moladar	Non-ACT Antimalarial	
Other ACT (e.g Duocotexin)	ACT	ACT
Other Antimalarial	Non-ACT Antimalarial	
Amoxyl	Amoxyl, Septrin, Cipro/Norflo	Antibiotic
Septrin	Amoxyl, Septrin, Cipro/Norflo	Antibiotic
Ciprofloxacin/Norfloxaccin	Amoxyl, Septrin, Cipro/Norflo	Antibiotic
Other Antibiotic	Other Antibiotic	Antibiotic
Panadol/Brufen/Hedex/Painkiller/Action/Maramoja	Painkiller	
Allergy/Cough/Asthma	Allergy/Cough/Asthma	
Antifungal	Antifungal	
Deworm	Deworm	
Stomach/GI	Stomach/GI	
Traditional/Herbal	Traditional/Herbal	
Vitamin	Vitamin	
Other	Other	
Don't know/Don't Remember		

Secondary Outcomes

Took ACT*:* From the question "Which medicines did you take," we defined a binary outcome for taking Coartem/Artefan/other ACT,

Source of Quality Approved ACT

Individuals who indicated that they had taken ACT were then asked, "Where was the ACT obtained?". Options given were "Used drugs at home; Pharmacy, with a prescription; shop or pharmacy without prescription; community health worker (CHW); neighbor; private health facility; government health facility; general store; and other. Individuals could select multiple options as they may have received multiple prescriptions or partially purchased the drug from a combination of sources. Therefore, categories for source of ACT are not mutually exclusive. Categories were further collapsed into these categories: home, health facility, shop or pharmacy without prescription, shop, or pharmacy with prescription, and other. Under the category of health facility, we further divided answer choices by type of facility with options of public facility and private facility.

Location of Testing

Finally, individuals who had indicated that they took a malaria test were asked "Where was the malaria test done?" and provided with options of: CHW (intervention only), shop or pharmacy, private clinic/hospital, government dispensary, government hospital, government health center, private lab, and other. For generalizability, categories were collapsed into 4 options +: no testing, testing at the CHW (intervention only), testing at a public facility, and testing at a private facility.
